# No Causal Effect of Telomere Length on Ischemic Stroke and Its Subtypes: A Mendelian Randomization Study

**DOI:** 10.3390/cells8020159

**Published:** 2019-02-14

**Authors:** Weijie Cao, Xingang Li, Xiaoyu Zhang, Jie Zhang, Qi Sun, Xizhu Xu, Ming Sun, Qiuyue Tian, Qihuan Li, Hao Wang, Jiaonan Liu, Xiaoni Meng, Lijuan Wu, Manshu Song, Haifeng Hou, Youxin Wang, Wei Wang

**Affiliations:** 1 Beijing Key Laboratory of Clinical Epidemiology, School of Public Health, Capital Medical University, Beijing 100069, China; wjcao@ccmu.edu.cn (W.C.); hydzxy@126.com (X.Z.); zhangjie@ccmu.edu.cn (J.Z.); cmusunqi@163.com (Q.S.); ming.s1204@outlook.com (M.S.); qiuyue__t@163.com (Q.T.); liqihuankk@163.com (Q.L.); hao.wang@ecu.edu.au (H.W.); liujiaonan9999@163.com (J.L.); mengxiaoni385@163.com (X.M.); wujuan811017@163.com (L.W.); songms@ccmu.edu.cn (M.S.); 2 School of Medical and Health Sciences, Edith Cowan University, Perth 6027, Australia; xingang.li@ecu.edu.au (X.L.); hfhou@163.com (H.H.); 3 School of Public Health, Taishan Medical University, Taian 271016, China; xzxu@tsmc.edu.cn

**Keywords:** Mendelian randomization study, single nucleotide polymorphisms, instrumental variable, ischemic stroke, telomere length

## Abstract

Background: Epidemiological studies observing inconsistent associations of telomere length (TL) with ischemic stroke (IS) are susceptible to bias according to reverse causation and residual confounding. We aimed to assess the causal association between TL, IS, and the subtypes of IS, including large artery stroke (LAS), small vessel stroke (SVS), and cardioembolic stroke (CES) by performing a series of two-sample Mendelian randomization (MR) approaches. Methods: Seven single nucleotide polymorphisms (SNPs) were involved as candidate instrumental variables (IVs), summarized from a genome-wide meta-analysis including 37,684 participants of European descent. We analyzed the largest ever genome-wide association studies of stroke in Europe from the MEGASTROKE collaboration with 40,585 stroke cases and 406,111 controls. The weighted median (WM), the penalized weighted median (PWM), the inverse variance weighted (IVW), the penalized inverse variance weighted (PIVW), the robust inverse variance weighted (RIVW), and the Mendelian randomization-Egger (MR-Egger) methods were conducted for the MR analysis to estimate a causal effect and detect the directional pleiotropy. Results: No significant association between genetically determined TL with overall IS, LAS, or CES were found (all *p* > 0.05). SVS was associated with TL by the RIVW method (odds ratio (OR) = 0.72, 95% confidence interval (CI): 0.54–0.97, *p* = 0.028), after excluding rs9420907, rs10936599, and rs2736100. Conclusions: By a series of causal inference approaches using SNPs as IVs, no strong evidence to support the causal effect of shorter TL on IS and its subtypes were found.

## 1. Introduction

According to the World Health Organization, in 2016 stroke was the second leading cause of death worldwide and a major contributor to disability, with ischemic stroke (IS) blocked blood vessels accounting for 87% of all stroke cases, compared to hemorrhagic stroke (HS) bleeding blood vessels in the brain accounting for 13% [1]. IS is a complex syndrome triggered by embolisms from the heart, artery-to-artery embolism, and in situ small vessel disease [2], finally leading to severely reduced blood flow and brain damage. The major etiological subtypes of IS are large artery stroke (LAS), cardioembolic stroke (CES), and small vessel stroke (SVS) [3]. The pathogenesis of IS is complicated and involves many different predisposing factors. With the ongoing demographic changes of aging of the population and health transitions, IS remains a major global health problem and its significance is predicted to increase in the future. While modifiable risk factors such as hypertension, obesity, smoking, physical inactivity, inflammatory disorders, and infections are believed to contribute to the disease risk, interventions have primarily focused on preventing the environmental interactions with genetic factors.

Telomeres are genetic DNA-protein complexes that include TTAGGG nucleotide repeats at the end of eukaryotic chromosomes, interacting with the environment to maintain the integrity and stability of the genome during cellular replication. Shorter telomeres are shown to be associated with increased all-cause mortality risk in the general population [4,5], resulting from cellular dysfunction, senescence and death [6,7]. Telomere dysfunction, a biomarker of cellular aging, may contribute to vascular aging [8]. Vascular aging is characterized by a gradual change of structure and function resulting in increased arterial stiffening. Oxidative stress and vascular inflammation are the principal drivers in endothelial dysfunction and atherosclerosis. They are also supposed to cause accelerated telomere attrition and subsequent premature cellular senescence in endothelial cells, vascular smooth muscle cells, and blood leukocytes [9]. In patients with an acute coronary syndrome, telomere loss has been reported to be associated with the risk of highly unstable atherosclerotic plaques and increased pro-inflammatory activity [9]. Pulse wave velocity, which increases with arterial stiffness, is the finite speed at which a heart contraction generating a pressure wave propagates through the circulatory system [10]. A previous study found differential relationships between aortic pulse wave velocity and telomere length (TL) in different age subjects [11]. In younger subjects (aged < 30 years), TL was significantly shorter among people with high aortic pulse wave velocity. By contrast, in those aged > 50 years there was a positive association with longer TL and high aortic pulse wave velocity. This indicates that the links between cellular senescence and vascular aging reflect complex regulatory mechanisms acting over the life-course.

The role of TL on vascular aging suggests that TL may potentially impact the onset of IS subtypes through atherosclerosis. In terms of LAS, it occurs when thrombosis or embolism blocks blood flow of brain vascular with atherosclerotic plaque and decreases the blood supply to that part of the brain [12]. SVSs are small infarcts resulting from the occlusion of penetrating branches of cerebral arteries that provide blood to the brain’s deep structures [13]. SVS is characterized pathologically by lipohyalinosis, arteriolosclerosis, and atherosclerosis (microatheroma). Endothelial dysfunction may be the major pathogenic mechanism for SVS, as to CES, for which risk factors include atrial fibrillation and aortic arch atheroma. Aging and multiple vascular risk factors result in an abnormal atrial substrate or atrial cardiopathy via a complex thrombo-genesis pathway, further increasing the risk of CES [14]. A large and rapidly expanding quantity of literature has reported the associations between TL and IS [15,16]. These observational studies, however, have consistently shown a controversial unexplainable inverse relationship between TL and IS [17,18]. To date, the causal effect between TL and IS remains unknown; thus, an urgent need to investigate the causal relationship for improving the strategies of stroke prevention and management is required. 

Mendelian randomization (MR) is fast becoming a popular method to investigate the causality from routinely conducted observational studies using genetic variations as a natural experiment and instrumental variables (IVs) to avoid the conventional bias of reverse causation and residual confounding [19]. A recent MR study [20] showed that shorter TL was marginally associated with decreased risk of stroke, which is inconsistent with previous studies. Furthermore, the role of shortened telomeres on coronary heart disease may act via fasting insulin level as a mediator based on MR analysis [21]. Type 2 diabetes may be causally associated with LAS [22].

In the present study, we performed a series of multiple-instrument two-sample MR analyses with summarized data from published genome-wide association studies (GWASs) to decipher the causal role of TL in IS subtypes, and to provide insight into potential mechanisms.

## 2. Materials and Methods

### 2.1. Data Source and Single Nucleotide Polymorphism(SNP)Selection

From a GWAS of TL in the ENGAGE Telomere Consortium [23], based on 37,684 individuals of European ancestry, we selected seven SNPs in seven loci that were associated with TL at a genome-wide significance threshold (*p* < 5 × 10^−8^). The association estimate for the “short” allele (in terms of per standard deviation decrease in TL per allele), standard error, and *p*-value for each SNP were obtained. The proportion of variance in average TL explained by individual SNPs ranged from 0.08% to 0.36%.

Data of seven TL-related SNPs with IS and its subtypes were retrieved as summary statistics from 438,847 European-descent individuals (40,585 cases; 406,111 controls) included in the MEGASTROKE collaboration [24]. The Trial of ORG 10172 in Acute Stroke Treatment criteria was used for IS subtyping, identifying 4373 LAS cases, 7193 CES cases, and 5386 SVS cases. Brain imaging was used to reconfirm that all cases were IS.

All participants provided written informed consent in all these corresponding original studies. Each study included in the stroke consortia was approved by the local institutional review board and ethics committee.

### 2.2. Statistical Analysis

MR is a type of instrumental variable analysis using genetic variants, such as SNPs, as proxies for risk factors of interests. MR assumes that SNPs are randomly distributed in the general population according to Mendel’s laws of inheritance (segregation, independent assortment), mimicking a randomization process, and that SNPs always precede the onset of disease, and thus could eliminate reverse causality. Three important assumptions need to be proved to ensure a valid IV within the MR analysis process [25]: (1) the genetic variants used as IVs are truly predictive of TL; (2) the genetic variants are not associated with measured and unmeasured confounders that influence both TL and stroke; and (3) the genetic variants affect stroke through their effects on TL only and not through any alternative causal pathways. 

The standard application of MR is a one-sample MR, which is performed within one population set containing complete data on the SNPs, exposure, and outcome for all participants [26]. Due to the rareness of comprehensive data in one single cohort, a two-sample MR is developed to allow analysis conducted in two separate samples—one for the exposure of interest and the other for the outcome [25]. In this study, we conducted six two-sample MR methods using “MendelianRandomization” R package [27], including the weighted median (WM), the penalized weighted median (PWM), the inverse variance weighted (IVW), the penalized inverse variance weighted (PIVW), the robust inverse variance weighted (RIVW), and the Mendelian randomization-Egger (MR-Egger) methods [28].

According to the advantages of each MR approach, these six methods can complement each other to provide a more reliable causal effect for our study. The inverse variance weighted methods are conventionally used in two-sample MR analysis. The MR-Egger methods are able to provide estimates of the casual effect, as well as to assess the directional pleiotropy under the weaker assumption, i.e., the “Instrument Strength Independent of Direct Effect (InSIDE)” assumption. The median-based methods could give consistent estimates when 50% or more of the genetic variants are valid IVs, which may be more appropriate than the MR-Egger methods in the existence of outlier genetic variants. The weighted methods provide more precise causal estimates based on more weight to the analysis. The penalized methods contribute to avoid the influence of pleiotropic effect in casual inference by omitting part of the genetic variants from the analysis. The robust methods make consistent estimates of causal effect under weaker assumptions than those of a conventional MR analysis. 

In the current study, we primarily estimated the effect of TL on IS and its subtypes using the conventional IVW method [29], which is an appropriate approach using summarized data from GWAS (individual-level data were not available). First, we conducted inverse variance weighting average of SNP-specific association with fixed effects in all types of IS other than CES. This analysis gives biased estimates if some of the instruments are invalid in the assumptions for causal inference based on the MR analysis [30]. Then, we addressed the first assumption (a true association between SNPs and TL) by selecting SNPs that strongly predicted TL. Genetic variants as proxies for TL were likely to satisfy the second assumption (no confounders existing). 

To assess the potential violation of the third assumption, the MR-Egger method was performed to confirm no directional pleiotropy if there is no intercept term. Scatter plots were then created to investigate the potential pleiotropy visually by presenting the associations between each SNP and the IS risk against associations with TL. Sensitivity analyses were performed to evaluate the robustness of results using WM, PWM, PIVW, RIVW, and MR-Egger methods. Finally, a power analysis using a web-based application was conducted (http://cnsgenomics.com/shiny/mRnd/) [31] to estimate the minimum detectable magnitude of association for IS, in terms of odds ratio (OR) per standard deviation of TL. The estimation of the detectable OR was obtained after specifying 80% power with 5% significance and by assuming that the variance in TL explained by the seven SNPs was 0.01 or 0.02, respectively.

All statistical tests were two-sided, and the evidence of association was declared at a pre-specified *p*-value below 0.05. The analyses were conducted using R version 3.3.3 and Stata/MP13.1 (Stata/MP, Texas, USA).

## 3. Results

### 3.1. Association of Genetic Variables with TL

**Figure 1:**
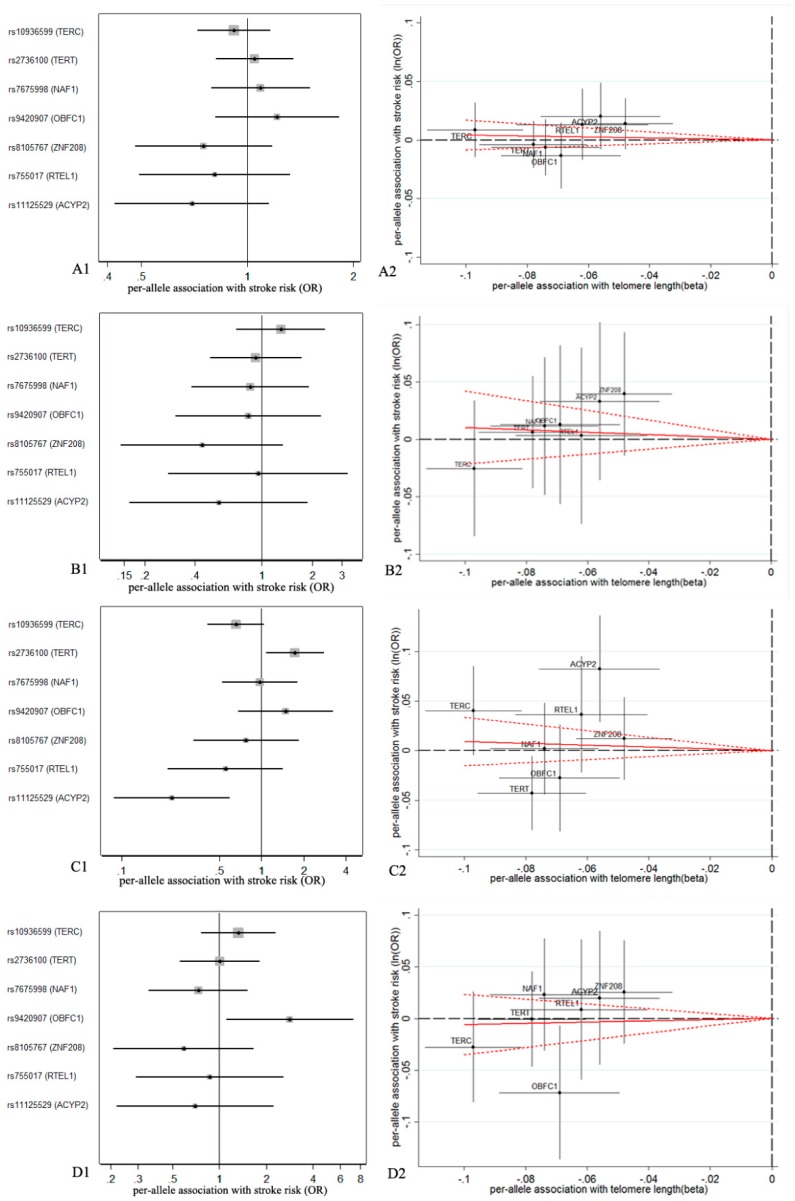
Forest plots and scatter plots of associations between telomere length (TL)-related single nucleotide polymorphisms (SNPs) and risk for all types and subtypes of ischemic stroke (IS). All ischemic stroke: (**A1**,**A2**); large artery stroke: (**B1**,**B2**); cardioembolic stroke: (**C1**,**C2**); small vessel stroke: (**D1**,**D2**). Forest plots (**A1**,**B1**,**C1**,**D1**) show the odds ratio (OR) with a horizontal line representing 95% confidence intervals (CIs) for the ‘short telomere’-associated SNP allele for stroke risk. Scatter plots (**A2**,**B2**,**C2**,**D2**) show the per-allele association with stroke risk plotted against the per-allele association with one standard deviation of TL (with vertical and horizontal black lines showing the 95% CI for each SNP). The slope of the red solid line (with dotted lines showing the 95% CI) in the scatter plots corresponds to the Mendelian randomization (MR) estimate.

Summarized data extracted from reported lectures are given in Table 1. We estimated the associations between each TL-related SNP and risk for IS and the subtypes, shown as forest plots in Figure 1. Two of seven SNPs used as IVs in the MR analyses demonstrated that TL was associated with CES (*p* = 0.023 for rs2736100 and *p* = 0.003 for rs11125529), however, the association had a different direction. Genetic prediction of shorter TL using SNP rs9420907 as a proxy was associated with SVS with statistical significance (*p* = 0.029).

### 3.2. Association of TL with IS

The MR estimates of TL on IS using conventional MR analysis are presented in Table 2. The IVW estimate using all seven SNPs showed no clear association between shorter TL and stroke (all ischemic stroke (AIS), OR = 0.96, 95% confidence interval (CI): 0.85–1.09; LAS, OR = 0.90, 95% CI: 0.66–1.24; CES, OR = 0.82, 95% CI: 0.52–1.3VS, OR = 1.06, 95% CI: 0.76–1.48). 

The associations of TL with all stroke subtypes were consistent in the sensitivity analysis that used the WM, PWM, PIVW, and RIVW methods but not in the MR-Egger method (Figure S1). The MR-Egger intercept test suggested a potential directional pleiotropy (*p* = 0.054 for LAS, *p* = 0.080 for CES, *p* = 0.054 for CES, using all seven SNPs), which was also reflected in the scatter plots (Figure 1). After excluding rs9420907, rs10936599, and rs2736100, shorter TL was associated with SVS (OR = 0.72, 95% CI: 0.54–0.97, *p* = 0.028) using the PIVW approach. However, we observed no such association for the other IS subtypes after excluding rs9420907 for LAS, and excluding rs2736100 and rs9420907 for CES.

The statistical power analysis of this current MR study is given in Table S1. Based on the sample size of 438,847, our MR analysis would need to have over 80% power at an alpha rate of 5% to detect a statistically significant causal effect of a relative 14.5% decrease in overall IS risk per one standard deviation of TL; the corresponding estimates were 51.0%, 32.8%, and 33.5% relative reductions for LAS, CES, and SVS (i.e., ORs of 0.490, 0.672, and 0.665, respectively). 

## 4. Discussion

In the present study, we investigated the potential causal role of TL in the development of IS and its subtypes by performing a series of complementary MR methods. With genetic variants as proxies for TL, our study only showed a suggestive association between shorter TL and SVS risk based on PIVW after the exclusion of potential invalid SNPs. The results provided no strong evidence for a causal role of TL in overall IS and any other subtypes, including LAS, SVS, and CES.

The MR-Egger estimate was discordant with conventional results on IS and its subtypes, although the potential pleiotropic SNPs were excluded. It is possible for the MR-Egger estimate to be biased due to the violations of the InSIDE assumption [32]. The WM method, as an alternative pleiotropy-robust estimation strategy, provides a valid estimate if at least 50% of selected genetic variants are valid IVs [33]. Further, the WM method gives a consistent estimate with other penalized and robust approaches when a causal effect of shorter TL on SVS is explored. 

Despite previous cross-sectional studies showing an association between shorter TL and IS risk, such a relationship has not been firmly endorsed by evidence from prospective studies [33,34,35], which is consistent with our results regarding the role of TL in overall IS. The meta-analysis consisting of six prospective studies identified no evidence of an association between TL and IS risk [34]. Similar results were also found in a pool-analysis [35]. In contrast to a meta-analysis that aggregated data from 11 cohort studies, the association between TL and stroke was found to be significant in a pooled analysis, but was not found in subgroup analysis, in either the prospective or retrospective study subgroups [36]. 

Due to the different pathologies in IS subtypes and the different scales of effect sizes in different original studies, results of the meta-analysis may be less reliable. Two MR studies were performed to examine the association between TL and stroke [20,37]. The first MR study showed that shorter telomere is a protective factor with marginally decreased risk of stroke based on individual-level data and small sample size of 6426 cases [20]. The second study showed the lack of a clear association of TL on the risk of all IS subtypes [37]. These results are comparable with our finding in all subtypes except SVS, whereas the estimates in our study were based on a larger sample size of 40,585 cases.

There were several published studies on TL and IS subtypes [11,15,38]. A prospective study found that shorter TL is associated with LAS, but is not statistically associated with SVS [15]. The association between shorter TL and increased CES risk was also reported in a retrospective study [38]. In contrast, we observed a potential association between TL shortening and decreased risk for SVS, and a null relationship of TL with LAS and CES. Learning that the complex association between aortic pulse wave velocity (as a biomarker of arterial stiffness) and TL changes with age [11], the non-linear relationship between TL and IS subtypes may be limited to detect their association. Further, the null association between TL and CES was essentially unchanged, despite the inclusion of rs7675998, which showed an association with increased risk of CES [38]. Due to the complex reason of cardiogenic thrombus, the role of TL on CES should be analyzed by stratified analysis on locations of thrombus to reduce such biases. In addition, it was reported that lower general cognitive ability level was associated with shorter TL [39], and shorter TL was causally related to a higher risk of suffering from Alzheimer’s disease [40]. The findings of previous studies imply that our result pertaining to TL with SVS may involve the same pathogenesis of cerebral small vessel disease for SVS [41]. Therefore, the role of TL on SVS warrants further investigation.

Our MR study has several strengths. First, our study investigated the largest dataset included in the MEGASTROKE collaboration, with a total of 438,847 European-descent individuals (40,585 cases and 406,111 controls), which minimizes the influence of population stratification. Second, as a result of the MR design, we conducted six complementary MR methods to prevent the reverse causation bias and to reduce potential confounding. Third, the IVs used in our study were independent SNPs, which could maximally reduce the interference of linkage disequilibrium.

Potential limitations in our study include the third stringent assumption in MR analysis needing to be proved. Additionally, potential nonlinearity roles of TL on IS were unable to be explored as individual-level data were not available [42]. Our power estimation enabled us to detect low-to-moderate associations based on very large sample sizes, which is limited by a small fraction of the variation in TL explained by SNPs (1%) [43]. Furthermore, we could not confirm that the associations between SNPs and TL were consistent across multiple tissues due to a lack of TL-related GWAS data on specific tissue. However, leukocyte TL could warrant a surrogate marker of TL in other tissues because the relevance between different tissues in the same individual has been demonstrated [44]. Finally, we could not assess compensatory mechanisms which attenuate the estimates and thus lower precision.

## 5. Conclusions

Using TL-associated SNPs as instrumental variables extracted from the GWAS data consisting of large European-descent cohorts, our MR analyses do not support a causal effect of shorter TL (exposures) on IS or its subtypes (outcome variables). Our results suggest that shorter TL may be a potential causal risk factor for SVS, but there remains a lack of evidence for a causal role of TL for LAS and CES. Further MR studies using individual-level data based on larger sample sizes are required to delineate any potential nonlinearity between TL and IS risk and to identify the effects of TL across subclasses of IS subtypes.

## Figures and Tables

**Table 1:** Summarized data for the genetic variants associated with telomere length.

SNP	Chr.	Locus	‘Short’ Allele	‘Other’ Allele	Beta Estimate	SE	Discovery *p*
rs10936599	3	*TERC*	T	C	−0.097	0.008	2.5 × 10^−31^
rs2736100	5	*TERT*	A	C	−0.078	0.009	4.4 × 10^−9^
rs7675998	4	*NAF1*	A	G	−0.074	0.009	4.3 × 10^−16^
rs9420907	10	*OBFC1*	A	C	−0.069	0.010	6.9 × 10^−11^
rs8105767	19	*ZNF208*	A	G	−0.048	0.008	1.1 × 10^−9^
rs755017	20	*RTEL1*	A	G	−0.062	0.011	6.7 × 10^−9^
rs11125529	2	*ACYP2*	C	A	−0.056	0.010	4.5 × 10^−8^

Chr.: chromosome; SE: standard error; SNP: single nucleotide polymorphism.

**Table 2:** Odds ratios for the association between TL and IS and its subtypes using different methodological approaches and exclusions for pleiotropic SNPs.

Ischemic Stroke	IVW		RIVW		MR-Egger		
	OR (95% CI)	*p*	OR (95% CI)	*p*	OR (95% CI)	*p*	Intercept (*p*)
AIS	0.96 (0.85, 1.09)	0.535	0.96 (0.87, 1.07)	0.452	1.32 (0.73, 2.37)	0.354	−0.02 (0.277)
LAS							
ALL SNPs	0.90 (0.66, 1.24)	0.530	0.90 (0.62, 1.30)	0.569	3.45 (0.79, 15.07)	0.100	−0.10 (0.054)
6 SNPs ^1^	0.79 (0.54, 1.15)	0.210	0.84 (0.29, 2.39)	0.741	2.94 (0.34, 25.33)	0.327	−0.09 (0.224)
CES							
ALL SNPs	0.82 (0.52, 1.32)	0.417	0.91 (0.56, 1.46)	0.683	1.56 (0.17, 13.96)	0.691	−0.04 (0.080)
5 SNPs ^2^	0.76 (0.39, 1.46)	0.404	0.98 (0.53, 1.80)	0.941	9.44 (0.55, 163.04)	0.123	−0.15 (0.112)
SVS							
ALL SNPs	1.06 (0.76, 1.48)	0.722	1.03 (0.72, 1.48)	0.867	2.59 (0.59, 11.38)	0.208	−0.07 (0.054)
4 SNPs ^3^	0.72 (0.45, 1.16)	0.176	0.72 (0.54, 0.97)	0.028	1.13 (0.07, 18.38)	0.932	−0.03 (0.747)

IVW: the inverse variance weighted method; RIVW: the robust inverse variance weighted method; MR-Egger: the Mendelian randomization-Egger method; OR: odds ratio; CI: confidence interval; AIS: all ischemic stroke; LAS: large artery stroke; CES: cardioembolic stroke; SVS: small vessel stroke. ^1^: analysis excluding rs9420907; ^2^: analysis excluding rs2736100 and rs9420907; ^3^: analysis excluding rs9420907, rs10936599, and rs2736100.

## Supplementary Materials

The following are available online at http://www.mdpi.com/2073-4409/8/2/159/s1, Figure S1: Sensitivity analysis for the associations of genetically predicted TL with ischemic stroke and its subtypes; Table S1: Approximate detectable odds ratio (OR) per one standard deviation of telomere length.
