# Supplementary material for: No Causal Effect of Telomere Length on Ischemic Stroke and Its Subtypes: A Mendelian Randomization Study
Source: Cells. 2019 Feb 14;8(2):159. doi: 10.3390/cells8020159 (PMC6407010; doi:10.3390/cells8020159)
Supplement: Supplementary file 1 [file cells-08-00159-s001.pdf]

Table S1. Approximate detectable odds ratio (OR) per one standard deviation of telomere length.

Ischemic stroke	Sample size	Proportion cases	OR (R^2=0.01)	OR (R^2=0.02)
All ischemic stroke	438,847	0.09	0.855	0.896
Large artery stroke	301,663	0.01	0.490	0.640
Cardioembolic stroke	362,661	0.02	0.672	0.767
Small vessel stroke	348,946	0.02	0.665	0.762

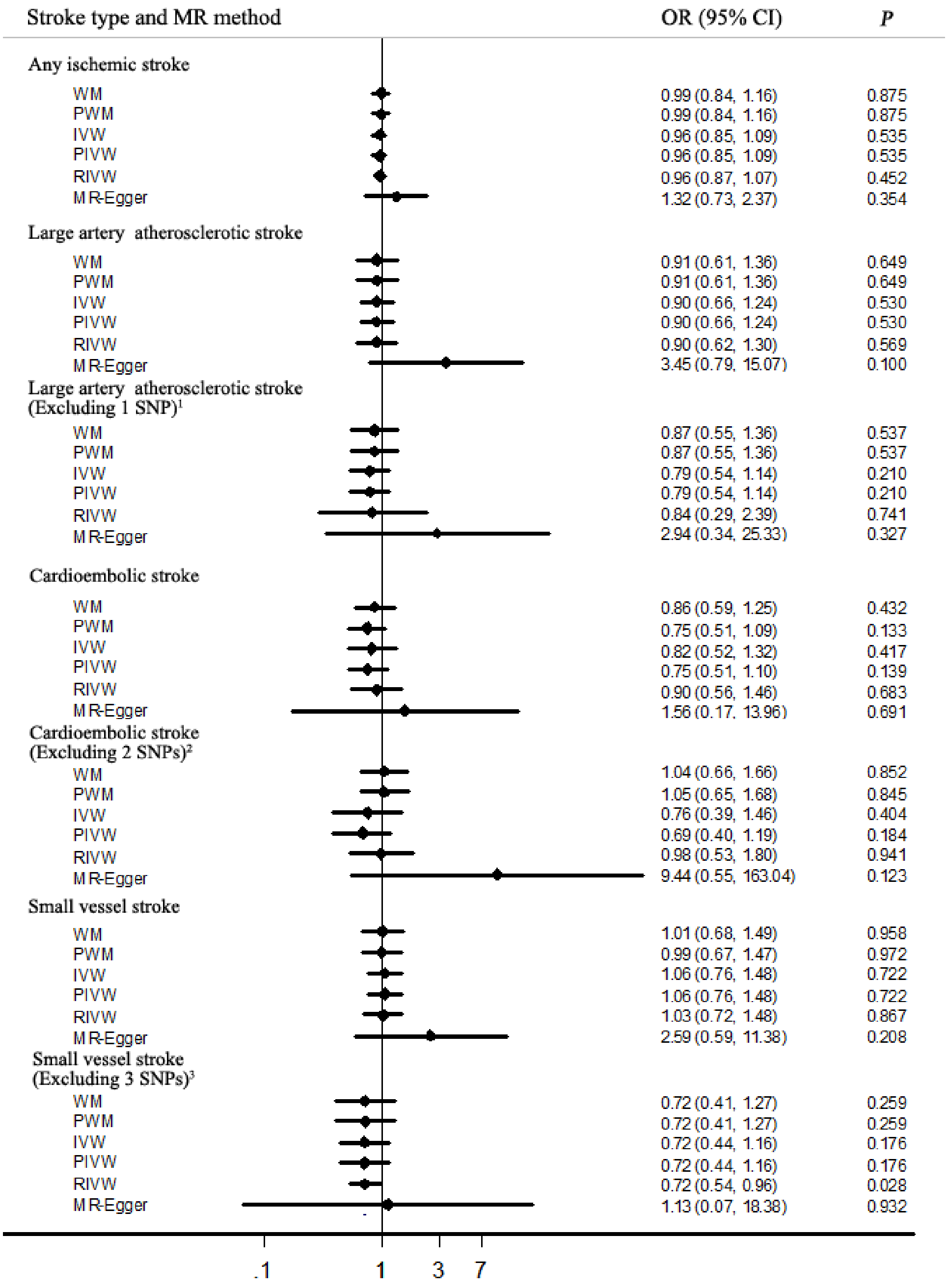

Figure S1. Sensitivity analysis for the associations of genetically predicted TL with IS and its subtypes. WM: the weighted median method; PWM: the penalized weighted median method; IVW: the inverse variance weighted method; PIVW: the penalized inverse variance weighted method; RIVW: the robust inverse variance weighted method.[1]: analysis excluding rs9420907; [2]: analysis excluding rs2736100 and rs9420907; [3]: analysis excluding rs9420907, rs10936599 and rs2736100.

Acknowledgments

Acknowledgement of the contributing studies and databases (MEGASTROKE project) that made GWAS summary data available. We gratefully acknowledge the assistance and contributions of Rainer Malik, Ganesh Chauhan, Matthew Traylor, Muralidharan Sargurupremraj, Yukinori Okada, Aniket Mishra, Loes Rutten-Jacobs, Anne-Katrin Giese, Sander W. van der Laan, Solveig Gretarsdottir, Christopher D. Anderson, Michael Chong, Hieab H. H. Adams, Tetsuro Ago, Peter Almgren, Philippe Amouyel, Hakan Ay, Traci M. Bartz, Oscar R. Benavente, Steve Bevan, Giorgio B. Boncoraglio, Robert D. Brown, Jr, Adam S. Butterworth, Caty Carrera, Cara L. Carty, Daniel I. Chasman, Wei-Min Chen, John W. Cole, Adolfo Correa, Ioana Cotlarciuc, Carlos Cruchaga, John Danesh, Paul I. W. de Bakker, Anita L. DeStefano, Marcel den Hoed, Qing Duan, Stefan T. Engelter, Guido J. Falcone, Rebecca F. Gottesman, Raji P. Grewal, Vilmundur Gudnason, Stefan Gustafsson, Jeffrey Haessler, Tamara B. Harris, Ahamad Hassan, Aki S. Havulinna, Susan R. Heckbert, Elizabeth G. Holliday, George Howard, Fang-Chi Hsu, Hyacinth I. Hyacinth, M. Arfan Ikram, Erik ingelsson, Marguerite R. Irvin, Xueqiu Jian, Jordi Jimenez-Conde, Julie A. Johnson, J. Wouter Jukema, Masahiro Kanai, Keith L. Keene, Brett M. Kissela, Dawn O. Kleindorfer, Charles Kooperberg, Michiaki Kubo, Leslie A. Lange, Carl D. Langefeld, Claudia Langenberg, Lenore J. Launer, Jin-Moo Lee, Robin Lemmens, Didier Leys, Cathryn M. Lewis, Wei-Yu Lin, Arne G. Lindgren, Erik Lorentzen, Patrik K. Magnusson, Jane Maguire, Ani Manichaikul, Patrick F. McArdle, James F. Meschia, Braxton D. Mitchell, Thomas H. Mosley, Michael A. Nalls, Toshiharu Ninomiya, Martin J. O'Donnell, Bruce M. Psaty, Sara L. Pulit, Kristiina Rannikmäe, Alexander P. Reiner, Kathryn M. Rexrode, Kenneth Rice, Stephen S. Rich, Paul M. Ridker, Natalia S. Rost, Peter M. Rothwell, Jerome I. Rotter, Tatjana Rundek, Ralph L. Sacco, Saori Sakaue, Michele M. Sale, Veikko Salomaa, Bishwa R. Sapkota, Reinhold Schmidt, Carsten O. Schmidt, Ulf Schminke, Pankaj Sharma, Agnieszka Slowik, Cathie L. M. Sudlow, Christian Tanislav, Turgut Tatlisumak, Kent D. Taylor, Vincent N. S. Thijs, Gudmar Thorleifsson, Unnur Thorsteinsdottir, Steffen Tiedt, Stella Trompet, Christophe Tzourio, Cornelia M. van Duijn, Matthew Walters, Nicholas J. Wareham, Sylvia Wassertheil-Smoller, James G. Wilson, Kerri L. Wiggins, Qiong Yang, Salim Yusuf, AFGen Consortium, Cohorts for Heart and Aging Research in Genomic Epidemiology (CHARGE) Consortium, International Genomics of Blood Pressure (iGEN-BP) Consortium, INVENT Consortium, STARNET, Joshua C. Bis, Tomi Pastinen, Arno Ruusalepp, Eric E. Schadt, Simon Koplev, Johan L. M. Björkegren, Veronica Codoni, Mete Civelek, Nicholas L. Smith, David A. Tregouet, Ingrid E. Christophersen, Carolina Roselli, Steven A. Lubitz, Patrick T. Ellinor, E. Shyong Tai, Jaspal S. Kooner, Norihiro Kato, Jiang He, Pim van der Harst, Paul Elliott, John C. Chambers, Fumihiko Takeuchi, Andrew D. Johnson, BioBank Japan Cooperative Hospital Group, COMPASS Consortium, EPiC-CVD Consortium, EPiC-interAct Consortium, International Stroke Genetics Consortium (ISGC), METASTROKE Consortium, Neurology Working Group of the CHARGE Consortium, NiNDS Stroke Genetics Network (SiGN), UK Young Lacunar DNA Study, MEGASTROKE Consortium, Dharambir K. Sanghera, Olle Melander, Christina Jern, Daniel Strbian, Israel Fernandez-Cadenas, W. T. Longstreth, Jr, Arndt Rolfs, Jun Hata, Daniel Woo, Jonathan Rosand, Guillaume Pare, Jemma C. Hopewell, Danish Saleheen, Kari Stefansson, Bradford B. Worrall, Steven J. Kittner, Sudha Seshadri, Myriam Fornage, Hugh S. Markus, Joanna M. M. Howson, Yoichiro Kamatani, Stephanie Debette, and Martin Dichgans
